# The histone demethylase JMJD1A induces cell migration and invasion by up-regulating the expression of the long noncoding RNA MALAT1

**DOI:** 10.18632/oncotarget.1785

**Published:** 2014-02-18

**Authors:** Andrew E. Tee, Dora Ling, Charlotte Nelson, Bernard Atmadibrata, Marcel E. Dinger, Ning Xu, Tamio Mizukami, Pei Y. Liu, Bing Liu, Belamy Cheung, Eddy Pasquier, Michelle Haber, Murray D. Norris, Takayoshi Suzuki, Glenn M. Marshall, Tao Liu

**Affiliations:** ^1^ Children's Cancer Institute Australia for Medical Research, Randwick, Sydney, Australia; ^2^ Garvan Institute of Medical Research, Darlinghurst, Sydney, Australia; ^3^ Graduate School of Bio-Science, Nagahama Institute of Bio-Science and Technology, Nagahama, Shiga, Japan; ^4^ Metronomics Global Health Initiative, Marseille, France; ^5^ Graduate School of Medical Science, Kyoto Prefectural University of Medicine, Taishogun Nishitakatsukasa-Cho, Kita-ku, Kyoto, Japan; ^6^ Kids Cancer Centre, Sydney Children's Hospital, Randwick, , Australia; ^7^ School of Women's & Children's Health, UNSW Medicine, University of New South Wales, Randwick, Sydney, Australia

**Keywords:** neuroblastoma, N-Myc, JMJD1A, histone demethylation, MALAT1

## Abstract

Patients with neuroblastoma due to N-Myc oncogene amplification have a high frequency of tumor metastasis. However, it is not clear how N-Myc induces cell migration, invasion and metastasis. The histone demethylase JMJD1A activates gene transcription by demethylating the lysine 9 residue of histone H3 (H3K9) at target gene promoters. The long noncoding RNA MALAT1 induces lung cancer cell migration and plays a pivotal role in lung cancer metastasis. Here we demonstrated that N-Myc up-regulated the expression of JMJD1A in N-Myc oncogene-amplified human neuroblastoma cells by directly binding to the JMJD1A gene promoter. Affymetrix microarray studies revealed that the gene second most significantly up-regulated by JMJD1A was MALAT1. Consistent with this finding, RT-PCR and chromatin immunoprecipitation assays showed that JMJD1A bound to the MALAT1 gene promoter and demethylated histone H3K9 at the MALAT1 gene promoter. Moreover, JMJD1A and MALAT1 induced, while the small molecule JMJD1A inhibitor DMOG suppressed, neuroblastoma cell migration and invasion. Taken together, our data identify a novel pathway through which N-Myc causes neuroblastoma cell migration and invasion, and provide important evidence for further development of more potent JMJD1A/MALAT1 inhibitors for the prevention of tumor metastasis.

## INTRODUCTION

Neuroblastoma, which originates from precursor neuroblast cells in the sympathetic nervous systems, is the most common extracranial solid tumor in children. *MYCN* oncogene amplification and consequent N-Myc mRNA and protein over-expression, are seen in a quarter of tumors and correlate with poorer prognosis in neuroblastoma patients [1, 2].

Myc oncoproteins, including N-Myc and c-Myc, induce malignant transformation and tumor progression by directly binding to cognate DNA sequences and modulating gene transcription [3, 4]. Myc oncoproteins activate gene transcription by directly binding to Myc-responsive element E-Boxes at target gene promoters.

Gene transcription is a dynamic process, during which lysine residues of histone H3 are modified by histone demethylases and methyltransferases to change RNA polymerase's ability to access the transcription start site [5, 6]. Many lines of evidence suggest that demethylation of repressive histone methylation marks such as histone H3 lysine 9 (H3K9) by histone demethylases is a prerequisite for transcriptional activation by transcription factors [7-9].

Also known as KDM3A and JHDM2A, JMJD1A belongs to the Jumonji C-domain-containing protein (JMJD) family, and demethylates mono-methyl and di-methyl histone H3K9 *in vitro* and *in vivo* [7-9]. While JMJD1A gene expression is up-regulated by androgen receptor activation [10], JMJD1A demethylates histone H3K9 at promoter regions of androgen receptor target genes, functions as a co-activator for androgen receptor, and induces transcriptional activation of androgen receptor target genes [9]. Similarly, whereas JMJD1A gene expression is up-regulated by β-adrenergic agonists, JMJD1A directly binds to promoter regions of β-adrenergic agonist target genes such as Ucp1, demethylates histone H3K9 at the promoters, and activates gene transcription [7].

The long noncoding RNA MALAT1, also known as NEAT2, is over-expressed in metastatic, compared with primary, lung cancer tissues, and is associated with poor prognosis in patients with non-small cell lung cancer [11]. Recent studies show that knocking-down MALAT1 expression impairs lung adenocarcinoma cell mobility and metastasis, suggesting the important role of MALAT1 in lung cancer metastasis [12, 13].

In the current study, we identified one Myc-responsive element E-Box at the JMJD1A gene core promoter, and showed that N-Myc up-regulated JMJD1A gene transcription by binding to JMJD1A gene promoter. JMJD1A demethylated histone H3K9 at the MALAT1 gene promoter, leading to transcriptional activation of MALAT1. These mechanisms contributed to neuroblastoma cell migration and invasion, which could be reversed by the small molecule JMJD1A inhibitor DMOG.

## RESULTS

### N-Myc up-regulates JMJD1A gene expression by directly binding to its gene promoter

**Figure 1:**
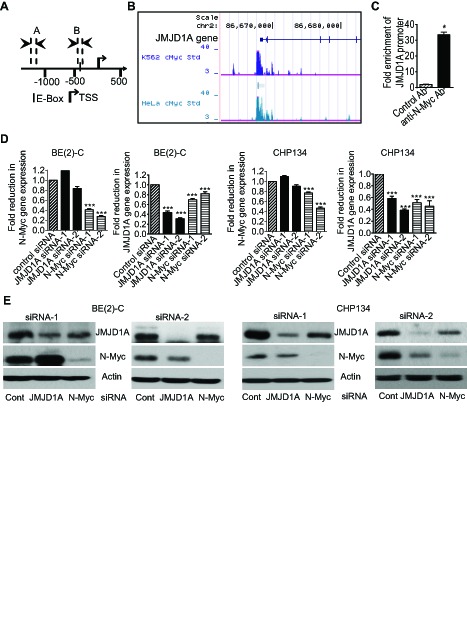
N-Myc up-regulates JMJD1A gene expression by directly binding to the JMJD1A gene promoter (A) Schematic representation of the Myc responsive element E-Box at the human JMJD1A gene core promoter. TSS represented transcription start site, and│represented the E-Box. Amplicon A and Amplicon B represented the up-stream control region and the E-Box-containing region respectively. (B) ChIP-Seq data from Dr. Michael Snyder's group at Yale University for the ENCODE/SYDH project generated from K562 and HeLa cells. (C) ChIP assays were performed with a control or anti-N-Myc antibody (Ab) and primers targeting Amplicon B and Amplicon A in BE(2)-C cells. Fold enrichment of the JMJD1A gene core promoter by the control or anti-N-Myc antibody was calculated by dividing PCR products from primers targeting the JMJD1A gene core promoter (Amplicon B) by PCR products from primers targeting the up-stream negative control region (Amplicon A). Fold enrichment by control antibody was artificially set as 1.0. (D-E) BE(2)-C and CHP134 neuroblastoma cells were transfected with scrambled control (Cont) siRNA, N-Myc siRNA-1, N-Myc siRNA-2, JMJD1A siRNA-1 or JMJD1A siRNA-2 for 48 hours, followed by RNA and protein extraction, real-time RT-PCR and immunoblot analyses of N-Myc and JMJD1A mRNA (D) and protein expression (E). Error bars represented standard error. * indicated *p* < 0.05, and *** indicated *p* < 0.001.

By screening human histone demethylase gene promoter regions with GenoMatix software, we found one Myc-responsive element E-box -420bp upstream of the JMJD1A gene transcription start site (Fig. 1A). We then examined a c-Myc chromatin immunoprecipitation-sequencing (ChIP-Seq) dataset, which was generated by Dr. Michael Snyder's group at Yale University for the ENCODE/SYDH project. As shown in Fig. 1B, the ChIP-seq data showed that the c-Myc oncoprotein bound to the JMJD1A gene core promoter region encompassing the E-Box in K562 and HeLa cells. Consistently, our own ChIP assays showed that an anti-N-Myc antibody efficiently immunoprecipitated the region of the JMJD1A gene core promoter carrying the E-box in BE(2)-C neuroblastoma cells (Fig. 1C). We next examined possible modulation of JMJD1A expression by N-Myc. As shown in Fig. 1D and Fig. 1E, transfection with N-Myc siRNA No.1 (N-Myc siRNA-1) or No.2 (N-Myc siRNA-2) reduced N-Myc mRNA and protein expression, and transfection with JMJD1A siRNA-1 and JMJD1A siRNA-2 knocked down JMJD1A mRNA and protein expression in *MYCN*-amplified BE(2)-C and CHP134 human neuroblastoma cells. Importantly, N-Myc siRNA-1 and N-Myc siRNA-2 significantly reduced JMJD1A mRNA and protein expression in the two neuroblastoma cell lines (Fig. 1D and Fig. 1E). Taken together, our data suggest that N-Myc up-regulates JMJD1A expression by directly binding to its gene core promoter.

### N-Myc modulates target gene expression partly through up-regulating JMJD1A gene expression

JMJD1A exerts biological effects by demethylating mono-methyl and di-methyl histone H3K9 and consequently modulating gene transcription [7-9]. We therefore performed differential gene expression studies with Affymetrix microarray in BE(2)-C cells 30 hours after transfection with scrambled control, JMJD1A siRNA-1 or N-Myc siRNA-1. Microarray data were subjected to normalization, summarization, annotation and differential gene expression analysis in R (http://www.r-project.org/) with bioconductor package (http://www.bioconductor.org/). We used 1.5 fold change as the cut-off for JMJD1A siRNA-1 versus control siRNA experiment and 2.0 fold change as the cut-off for N-Myc siRNA-1 versus control siRNA experiment, since JMJD1A siRNA-1 caused less changes in gene expression in 30 hours. The genome-wide analysis revealed that JMJD1A siRNA-1 reduced the expression of 0.15% (63 probes/41717 probes) of genes, but up-regulated the expression of 0.67% (280 probes/41717 probes) of genes (Supplementary Table S1). In comparison, the genome-wide analysis revealed that N-Myc siRNA-1 reduced the expression of 1.22% (260 probes/21255 probes) of genes, but up-regulated the expression of 1.40% (298 probes/21255 probes) of genes (Supplementary Table S2).

**Figure 2:**
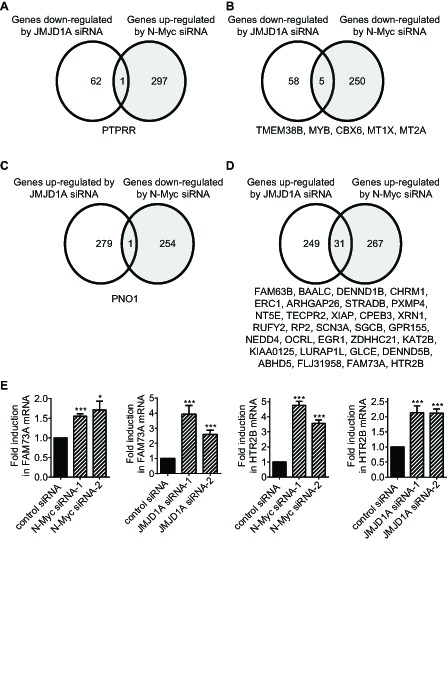
N-Myc and JMJD1A commonly up-regulate and commonly down-regulate the expression of subsets of genes (A-D) Genes down-regulated by JMJD1A siRNA-1 but up-regulated by N-Myc siRNA-1 (A), genes commonly down-regulated by JMJD1A siRNA-1 and N-Myc siRNA-1 (B), genes up-regulated by JMJD1A siRNA-1 but down-regulated by N-Myc siRNA-1 (C), and genes commonly up-regulated by JMJD1A siRNA-1 and N-Myc siRNA-1 (D), were identified by Affymetrix microarray analysis in BE(2)-C neuroblastoma cells 30 hours after siRNA transfections. (E) BE(2)-C cells were transfected with control siRNA, N-Myc siRNA-1, N-Myc siRNA-2, JMJD1A siRNA-1 or JMJD1A siRNA-2, followed by RNA extraction and RT-PCR analysis of FAM73A and HTR2B gene expression. Error bars represented standard error. * and *** indicated p < 0.05 and 0.001 respectively.

Importantly, while 1.59% (1 probe/63 probes) of genes down-regulated by JMJD1A siRNA-1 were up-regulated by N-Myc siRNA-1 (Fig. 2A), 7.94% (5 probes/63 probes) of genes down-regulated by JMJD1A siRNA-1 were also down-regulated by N-Myc siRNA-1 (Fig. 2B). In comparison, while 0.36% (1 probe/280 probes) of genes up-regulated by JMJD1A siRNA-1 were down-regulated by N-Myc siRNA-1 (Fig. 2C), 11.07% (31 probes/280 probes) of genes up-regulated by JMJD1A siRNA-1 were also up-regulated by N-Myc siRNA-1 (Fig. 2D). To validate the Affymetrix microarray data, we performed RT-PCR analysis of FAM73A and HTR2B, which were up-regulated by both JMJD1A siRNA-1 and N-Myc siRNA-1 (Fig. 2D). RT-PCR studies confirmed that transfection with JMJD1A siRNA-1, JMJD1A siRNA-2, N-Myc siRNA-1 or N-Myc siRNA-2 significantly increased FAM73A and HTR2B mRNA expression in neuroblastoma cells (Fig. 2E). Taken together, the data suggested that JMJD1A and N-Myc commonly up-regulate the expression of a subset of genes and commonly down-regulate the expression of a subset of genes, and that N-Myc modulates target gene expression partly through up-regulating JMJD1A gene expression.

### JMJD1A up-regulates MALAT1 gene expression by demethylating histone H3K9 at the MALAT1 gene promoter

**Figure 3:**
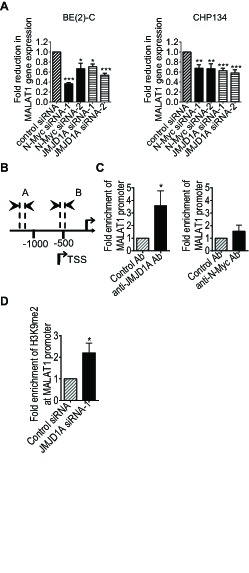
JMJD1A up-regulates MALAT1 gene transcription by demethylating histone H3K9 at the MALAT1 gene promoter (A) BE(2)-C and CHP134 cells were transfected with scrambled control, JMJD1A siRNA-1, JMJD1A siRNA-2, N-Myc siRNA-1 or N-Myc siRNA-2. MALAT1 RNA expression was analysed by real-time RT-PCR. (B) Schematic representation of the MALAT1 gene promoter region. (C) ChIP assays were performed in BE(2)-C cells with a control, anti-JMJD1A or anti-N-Myc antibody (Ab), and real-time PCR with primers targeting MALAT1 gene promoter region or upstream negative control region. Fold enrichment of the MALAT1 gene promoter by the control, anti-JMJD1A or anti-N-Myc antibody was calculated by dividing PCR products from primers targeting the MALAT1 gene promoter by PCR products from primers targeting negative control region. Fold enrichment by control antibody was artificially set as 1.0. (D) BE(2)-C cells were transfected with scrambled control siRNA or JMJD1A siRNA-1 for 48 hours, followed by ChIP assays with an anti-di-methyl-H3K9 (H3K9me2) antibody and real-time PCR with primers targeting MALAT1 gene promoter region or upstream negative control region. Fold change in the presence of H3K9me2 at the MALAT1 gene core promoter was obtained after dividing fold enrichment of H3K9me2 at the MALAT1 gene core promoter in JMJD1A siRNA-1 transfected samples by fold enrichment of H3K9me2 at the MALAT1 gene core promoter in control siRNA transfected samples. * indicated *p* < 0.05, ** *p* < 0.01 and *** *p* < 0.001.

As shown in Supplementary Table S1, the gene second most significantly reduced by JMJD1A siRNA-1 was the long noncoding RNA MALAT1. In comparison, MALAT1 expression was not reduced by N-Myc siRNA-1 in the Affymetrix microarray data (Supplementary Table S2) which were acquired from BE(2)-C neuroblastoma cells 30 hours after siRNA transfections. As N-Myc up-regulated JMJD1A expression (Fig. 1), we examined whether both JMJD1A siRNAs and N-Myc siRNAs reduced MALAT1 expression at a later time point. Real-time RT-PCR analysis showed that knocking-down JMJD1A or N-Myc expression with JMJD1A siRNA-1, JMJD1A siRNA-2, N-Myc siRNA-1 or N-Myc siRNA-2 for 48 hours all significantly reduced MALAT1 expression in both BE(2)-C and CHP134 cells (Fig. 3A).

JMJD1A is known to up-regulate gene transcription by demethylating mono-methyl and di-methyl histone H3K9 [7-9]. We therefore performed ChIP assays with a control IgG, anti-JMJD1A antibody or anti-N-Myc antibody and PCR with primers targeting the MALAT1 gene core promoter or a negative control region. Results showed that the anti-JMJD1A antibody, but not the anti-N-Myc antibody, efficiently immunoprecipitated the MALAT1 gene core promoter (Fig. 3B and Fig. 3C). To confirm that JMJD1A demethylated histone H3K9 at the MALAT1 gene promoter, we transfected BE(2)-C cells with control siRNA or JMJD1A siRNA-1 for 48 hours, followed by ChIP assays with a control IgG or an anti-di-methyl H3K9 antibody and PCR with primers targeting the MALAT1 gene core promoter or a negative control region. Results showed that JMJD1A siRNA-1 significantly increased the presence of di-methyl histone H3K9 at the MALAT1 gene promoter (Fig. 3D). Taken together, the data suggest that JMJD1A up-regulates MALAT1 gene expression by directly binding to the MALAT1 gene promoter, leading to histone H3K9 demethylation, and that N-Myc indirectly up-regulates MALAT1 expression by activating JMJD1A gene transcription.

### JMJD1A induces neuroblastoma cell migration and invasion

**Figure 4:**
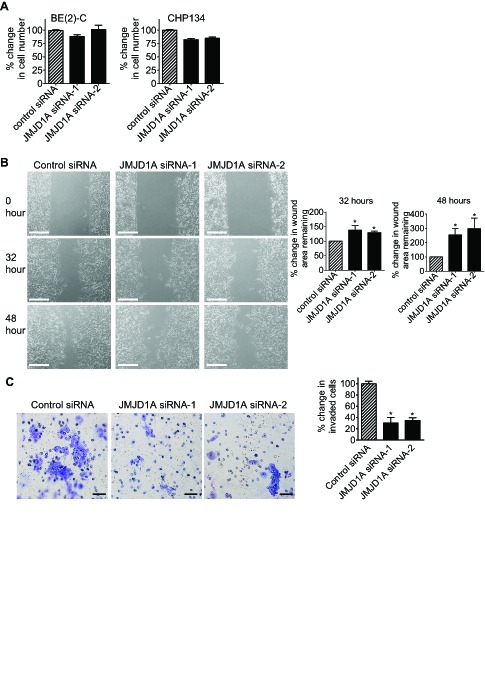
JMJD1A induces neuroblastoma cell migration and invasion (A) BE(2)-C and CHP134 cells were transfected with control siRNA, JMJD1A siRNA-1 or JMJD1A siRNA-2. Seventy-two hours later, the numbers of viable cells were analysed by Alamar blue assays. (B) Ibidi Culture Inserts were placed in the center of 6 well plates. BE(2)-C cells were added into the wells outside the inserts, and transfected with control siRNA, JMJD1A siRNA-1 or JMJD1A siRNA-2. Sixteen hours later, the inserts were removed to create “wounds”. Photos of the “wounds” were taken under microscope and the area of the remaining “wounds” were measured and analyzed with Image J software. The area of the remaining “wounds” for control siRNA-transfected cells was artificially set as 100%. Scale bars represented 250 μm. (C) BE(2)-C cells were transfected with control siRNA, JMJD1A siRNA-1 or JMJD1A siRNA-2. Twenty hours later, cells were detached and added onto BD Falcon Cell Culture Inserts coated with Matrigel inside BD BioCoat Matrigel Invasion Chambers. Cells invaded through Matrigel to the bottom side of the inserts were stained, photographed under microscope and quantified. Scale bars represented 40 μm. Error bars represented standard error. * indicated *p* < 0.05.

We next examined whether JMJD1A contributed to a cancer phenotype. Alamar blue assays showed that knocking-down JMJD1A gene expression with JMJD1A siRNA-1 or JMJD1A siRNA-2 for 72 hours did not have a significant effect on BE(2)-C cell proliferation, but slightly reduced CHP134 cell proliferation (Fig. 4A). We therefore used BE(2)-C cells for cell migration and invasion assays. For cell migration assays, BE(2)-C cells were plated into 6 well plates with Ibidi Culture Inserts in the centre, and transfected with control siRNA, JMJD1A siRNA-1 or JMJD1A siRNA-2. Sixteen hours later, the inserts were removed to create “wounds”. Measurement of the area of the “wounds” showed that JMJD1A siRNAs, compared with control siRNAs, increased the area of the remaining “wounds” by approximately 30% and 150% respectively, 32 hours and 48 hours after the removal of the inserts (Fig. 4B). For cell invasion assays, BE(2)-C cells were transfected with control siRNA, JMJD1A siRNA-1 or JMJD1A siRNA-2 for 20 hours. After being detached, the cells were added onto Matrigel-coated BD Falcon Cell Culture Inserts inside BD BioCoat Matrigel Invasion Chambers. Quantification of cells which had invaded through Matrigel 22 hours later, showed that suppression of JMJD1A reduced neuroblastoma cell invasion by approximately 70% (Fig. 4C). The data suggest that JMJD1A induces neuroblastoma cell migration and invasion.

### MALAT1 induces neuroblastoma cell migration and invasion

**Figure 5:**
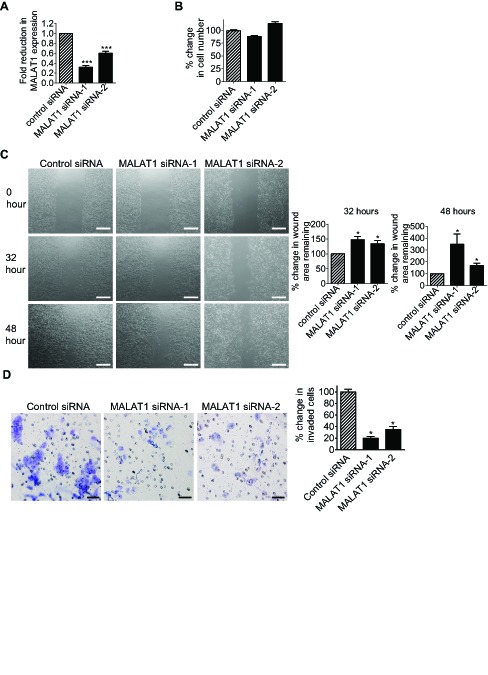
MALAT1 induces neuroblastoma cell migration and invasion (A) BE(2)-C cells were transfected with control siRNA, MALAT1 siRNA-1 or MALAT1 siRNA-2. Forty-eight hours later, RNA was extracted from the cells, and subjected to RT-PCR analysis of MALAT1 RNA expression. (B) BE(2)-C cells were transfected with control siRNA, MALAT1 siRNA-1 or MALAT1 siRNA-2. Sixty-four hours later, the numbers of viable cells were analysed by Alamar blue assays. (C) Ibidi Culture Inserts were placed in the center of 6 well plates. BE(2)-C cells were added into the wells outside the inserts, and transfected with control siRNA, MALAT1 siRNA-1 or MALAT1 siRNA-2. Sixteen hours later, the inserts were removed to create “wounds”. Photos of the “wounds” were taken under microscope, and the area of the remaining “wounds” were measured and analyzed with Image J software. The area of the remaining “wounds” for control siRNA-transfected cells was artificially set as 100%. Scale bars represented 250 μm. (D) BE(2)-C cells were transfected with control siRNA, MALAT1 siRNA-1 or MALAT1 siRNA-2. Twenty hours later, cells were detached and added onto BD Falcon Cell Culture Inserts coated with Matrigel inside BD BioCoat Matrigel Invasion Chambers. Cells invaded through Matrigel to the bottom side of the inserts were stained, photographed under microscope and quantified. Scale bars represented 40 μm. Error bars represented standard error. * indicated p < 0.05, and *** indicated p < 0.001.

We next examined whether MALAT1 also induced cell migration and invasion. RT-PCR and Alamar blue assays showed that transfection with MALAT1 siRNA-1 or MALAT1 siRNA-2 reduced MALAT1 gene expression (Fig. 5A), but did not have a significant effect on BE(2)-C cell proliferation (Fig. 5B). BE(2)-C cells were then plated into 6 well plates with Ibidi Culture Inserts in the centre, transfected with control siRNA, MALAT1 siRNA-1 or MALAT1 siRNA-2, and subjected to “wound” healing assays after the removal of the inserts. Measurement of the area of the “wounds” showed that MALAT1 siRNA-1 and MALAT1 siRNA-2, compared with control siRNA, increased the area of remaining “wounds” by 47% and 34% respectively thirty-two hours after the removal of the inserts, and by 248% and 68% respectively forty-eight hours after the removal of the inserts (Fig. 5C). For cell invasion assays, BE(2)-C cells were transfected with control siRNA, MALAT1 siRNA-1 or MALAT1 siRNA-2 for 20 hours, and then added onto Matrigel-coated BD Falcon Cell Culture Inserts inside BD BioCoat Matrigel Invasion Chambers. Quantification of cells which had invaded through the Matrigel 22 hours later, showed that MALAT1 siRNA-1 and MALAT1 siRNA-2 reduced BE(2)-C cell invasion by approximately 80% and 65% respectively (Fig. 5D). The data suggest that MALAT1 induces neuroblastoma cell migration and invasion.

### The small molecule JMJD1A inhibitor DMOG suppresses neuroblastoma cell migration and invasion

We next examined whether small molecule JMJD1A inhibitors suppressed neuroblastoma cell migration and invasion. We previously identified *N*-oxalylglycine (NOG) and its derivatives as histone demethylase inhibitors, and demonstrated that its dimethyl ester prodrug DMOG exerted histone lysine methylating activity in cells [14]. As DMOG was inactive outside cells and NOG could not permeate into cells, we analysed the selectivity and activity of NOG/DMOG against various histone demethylases *in vitro* by using NOG as the model compound. As shown in Table 1 and Supplementary Fig. S1, NOG selectively inhibited JMJD1A with an IC_50_ of 67μM, and inhibited other histone demethylases at much higher concentrations.

**Figure 6:**
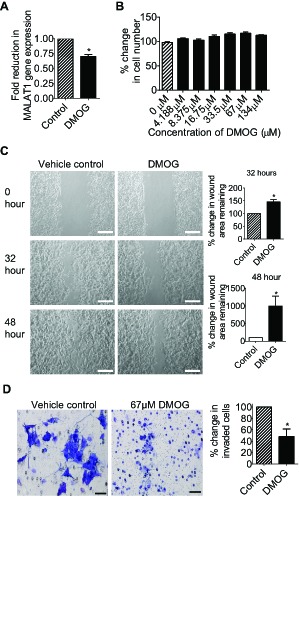
The JMJD1A inhibitor DMOG suppresses neuroblastoma cell migration and invasion (A) BE(2)-C cells were treated with vehicle control or 67μM DMOG. Forty-eight hours later, RNA was extracted from the cells, and subjected to RT-PCR analysis of MALAT1 RNA expression. (B) BE(2)-C cells were treated with a range of doses of DMOG. Seventy-two hours later, the numbers of viable cells were analysed by Alamar blue assays. (C) Ibidi Culture Inserts were placed in the center of 6 well plates. BE(2)-C cells were added into the wells outside the inserts, and treated with vehicle control or 67μM DMOG. Sixteen hours later, the inserts were removed to create “wounds”. Photos of the “wounds” were taken under microscope and the area of the remaining “wounds” were measured and analyzed with Image J software. The area of the remaining “wounds” for control siRNA-transfected cells was artificially set as 100%. Scale bars represented 250 μm. (D) BE(2)-C cells were treated with vehicle control or 67μM DMOG for 20 hours, and then detached and added onto BD Falcon Cell Culture Inserts coated with Matrigel inside BD BioCoat Matrigel Invasion Chambers. Cells invaded through Matrigel to the bottom side of the inserts were stained, photographed under microscope and quantified. Scale bars represented 40 μm. Error bars represented standard error. * indicated p < 0.05.

Next we examined the effects of DMOG on neuroblastoma cell proliferation, migration and invasion. As shown in Fig. 6A and Fig. 6B, treatment with 67μM DMOG reduced MALAT1 gene expression in BE(2)-C cells, and treatment with DMOG at a range of doses had no effect on cell proliferation. For cell migration assays, BE(2)-C cells were plated into 6 well plates with Ibidi Culture Inserts in the centre, and treated with vehicle control or 67μM DMOG after the removal of the inserts. “Wound” healing assays showed that DMOG increased the area of remaining “wounds” by approximately 47% and 895% respectively 32 hours and 48 hours after the removal of the inserts (Fig. 6C). For cell invasion assays, BE(2)-C cells were treated with vehicle control or 67μM DMOG for 20 hours, and then added onto Matrigel-coated BD Falcon Cell Culture Inserts inside BD BioCoat Matrigel Invasion Chambers. Quantification of cells which had invaded through the Matrigel 22 hours later, showed that DMOG reduced BE(2)-C cell invasion by approximately 52% (Fig. 6D). The data suggest that suppression of JMJD1A activity with the small molecule inhibitor DMOG can be used to reduce neuroblastoma cell migration and invasion.

## DISCUSSION

JMJD1A gene expression is down-regulated by the microRNA mir-155 [15], and up-regulated by hypoxia-inducible factor 1α (HIF1α), hypoxia, starvation and iron scavengers in tumor tissues [16-20]. Myc oncoproteins, including N-Myc and c-Myc, are well-known to exert oncogenic effects by binding to Myc-responsive element E-boxes at target gene promoters, leading to transcriptional activation [3], and by binding to Sp1-binding sites at target gene promoters, leading to transcriptional repression [21-23]. In this study, we have identified a canonical Myc-responsive element E-Box at the JMJD1A gene core promoter, and found that c-Myc oncoprotein binds to JMJD1A gene core promoter encompassing the E-Box in a publicly available ChIP-Seq dataset. Our own experiments have confirmed that N-Myc directly binds to JMJD1A gene core promoter and up-regulates JMJD1A mRNA and protein expression in neuroblastoma cells. Taken together, these data suggest that N-Myc oncoprotein up-regulates JMJD1A gene expression by directly binding to the JMJD1A gene core promoter.

As a histone demethylase, JMJD1A exerts biological effects by demethylating mono-methyl and di-methyl histone H3K9 and consequently modulating gene transcription [7-9]. While mono-methyl and di-methyl histone H3K9 at gene promoters are markers for transcriptional repression [24], di-methyl histone H3K9 has also been shown to be present at actively transcribed gene regions [25]. Our genome-wide differential gene expression study with Affymetrix microarray shows that knocking-down JMJD1A with siRNA leads to four times as many genes up-regulated than genes down-regulated in neuroblastoma cells. The result suggests that JMJD1A can function as a transcriptional activator and a repressor, but predominantly a transcriptional repressor in neuroblastoma cells. Furthermore, our genome-wide differential gene expression study confirms that JMJD1A and N-Myc commonly up- and down-regulate the expression of a subset of genes. As N-Myc up-regulates JMJD1A gene expression, the data suggest that N-Myc modulates gene expression partly through up-regulating JMJD1A expression.

One of the genes most significantly reduced by JMJD1A siRNAs in our Affymetrix microarray study is the long noncoding RNA MALAT1. Our RT-PCR and ChIP assays confirm that JMJD1A directly binds to the MALAT1 gene core promoter, and demethylates histone H3K9 at the MALAT1 gene core promoter. The data indicate that JMJD1A up-regulates MALAT1 gene expression by demethylating H3K9 at the MALAT1 gene core promoter. This is consistent with previous reports that JMJD1A activates gene transcription by demethylating histone H3K9 at promoter regions of target genes, such as transition protein-1, protamine 1 [8, 26], differentiation growth factor 15 [27], peroxisome proliferator-activated receptor α and mitochondrial uncoupling protein 1 [7]. Additionally, our RT-PCR and ChIP experiments show that, while N-Myc up-regulates MALAT1 gene expression, N-Myc protein does not bind to the MALAT1 gene promoter. This suggests that N-Myc up-regulates MALAT1 expression indirectly through modulating JMJD1A gene expression.

JMJD1A has been well documented to induce tumor progression by up-regulating HIF1α expression and stimulating angiogenesis [16, 18, 20, 27]. Recent studies show that JMJD1A is highly expressed in metastatic human prostate and colorectal cancer tissues, compared with primary tumors [16, 28]. Likewise, the long noncoding RNA MALAT1 is over-expressed in metastatic human lung, liver and colorectal cancer tissues [11, 29-31], and a high level of MALAT1 expression is associated with poor prognosis in patients with non-small cell lung cancer [11]. Additionally, recent studies show that MALAT1 induces liver and colorectal cancer cell migration and invasion in vitro [29, 31], and induces lung cancer cell migration, invasion and metastasis in vitro and in vivo [12, 13]. The current study demonstrates that JMJD1A exerts minor effects, while MALAT1 shows no effect, on neuroblastoma cell proliferation. Importantly, knocking-down JMJD1A and MALAT1 gene expression significantly reduces neuroblastoma cell migration and invasion. As N-Myc oncoprotein up-regulates JMJD1A expression and JMJD1A induces MALAT1 expression, our data suggest that N-Myc oncoprotein induces neuroblastoma cell migration and invasion through up-regulating JMJD1A expression, and that JMJD1A induces neuroblastoma cell migration and invasion through up-regulating MALAT1 expression.

Long noncoding RNAs exert biological effects mainly through regulating gene transcription in cis and in trans [32, 33]. Recent genome-wide differential gene expression and RT-PCR studies show that MALAT1 decreases the expression of the anti-metastatic MIA2 (melanoma inhibitory activity 2) and ROBO1 (roundabout 1), and up-regulates the expression of the pro-metastatic GPC6 (glypican 6), LPHN2 (latrophilin 2), CDCP1 (CUB domain containing protein 1) and ABCA1 (ATP-binding cassette, sub-family A, member 1) in lung cancer cells [13]. In this study, our Affymetrix microarray and RT-PCR data show that knocking-down JMJD1A and N-Myc gene expression commonly up-regulates HTR2B gene expression. As stable MALAT1 knockout in lung cancer cells up-regulates HTR2B expression [13] and silencing of HTR2B is a marker for ovarian tumor metastasis in ovarian cancer patients [34], we propose that repression of HTR2B expression contributes to MALAT1-mediated tumor cell migration, invasion and metastasis.

Histone deacetylase inhibitors are among the most promising novel anticancer agents [35], and have been approved by US Food and Drug Administration for cancer therapy. The current study shows that the small molecule JMJD1A inhibitor DMOG reduces MALAT1 gene expression, does not have an effect on neuroblastoma cell proliferation, but significantly reduces neuroblastoma cell migration and invasion. This is consistent with recent reports that repression of JMJD1A or MALAT1 with small hairpin RNAs or antisense oligonucleotides do not affect tumor cell proliferation, but reduce tumor cell migration, invasion and metastasis in vitro and in vivo [13, 27]. Our data indicate that more potent and selective JMJD1A inhibitors may be efficacious in blocking tumor cell migration, invasion and metastasis in cancer patients.

In summary, this study demonstrates that a novel pathway, involving transcriptional up-regulation of JMJD1A, which demethylates histone H3K9 at MALAT1 gene promoter and activates MALAT1 gene transcription, plays an important role in N-Myc oncoprotein-mediated neuroblastoma cell migration and invasion. Moreover, the small molecule JMJD1A inhibitor DMOG reduces MALAT1 expression and suppresses neuroblastoma cell migration and invasion. These findings therefore identify JMJD1A and MALAT1 as important factors for N-Myc-mediated neuroblastoma cell migration, invasion and potentially metastasis, and provide important evidence for further development of more selective and potent JMJD1A/MALAT1 inhibitors for the prevention of tumor metastasis.

## METHODS

### Cell culture

Neuroblastoma BE(2)-C and CHP134 cells were cultured in Dulbecco's modified Eagle's medium supplemented with 10% fetal calf serum.

### siRNA transfection

Cells were transfected with siRNAs from Qiagen (Qiagen, Hamburg, Germany) or Ambion (Ambion, Austin, TX, USA) using Lipofectamine 2000 (Invitrogen, Carlsbad, CA, USA) reagent as we described [22, 23]. The target sequences for N-Myc siRNAs were CCCGGACGAAGATGACTTCTA and CGTGCCGGAGTTGGTAAAGAA, JMJD1A siRNAs GCACAGTCCTCCATACGTT and GGAUGUAAACAGUCUUCGA, and MALAT1 siRNAs CACAGGGAAAGCGAGUGGUUGGU and GACAGGUAUCUCUUCGUUA.

### RT-PCR and immunoblot analyses

Gene expression in tumor cells was examined by quantitative real-time RT-PCR as described previously [21, 36]. For the analysis of protein expression by immunoblot, cells were lysed, protein extracted and separated by gel electrophoresis. After western transfer, membranes were probed with mouse anti-N-Myc antibody (1:1000) (Santa Cruz Biotech, CA, USA) or rabbit anti-JMJD1A antibody (1:500) (Abcam, Cambridge, MA, USA), followed by horseradish peroxidase-conjugated anti-mouse (1:10000) or anti-rabbit (1:20000) antiserum (Santa Cruz Biotech). Protein bands were visualized with SuperSignal (Pierce, Rockford, IL, USA). The membranes were lastly re-probed with an anti-actin antibody (Sigma, St Louis, MO, USA) as loading controls.

### Affymetrix microarray study

Neuroblastoma BE(2)-C cells were transfected with scrambled control siRNA, N-Myc siRNA-1 or JMJD1A siRNA-1. Thirty hours after transfection, RNA was extracted from the cells with RNeasy mini kit. Differential gene expression was examined with Affymetrix Arrays (Affymetrix, Santa Clara, CA, USA), according to the manufacturer's instruction. Results from the microarray hybridization were analysed in R (http://www.r-project.org/) with bioconductor package (http://www.bioconductor.org/).

### Cell proliferation assays

Cell proliferation was examined with Alamar blue assays [37]. Briefly, cells were plated into 96 well plates, transfected with various siRNAs or treated with different dosages of DMOG. Seventy-two hours later, cells were incubated with Alamar blue (Invitrogen) for 5 hours, and plates were then read on a micro-plate reader at 570/595 nm. Results were calculated according to the optical density absorbance units and expressed as percentage changes in cell numbers.

### Cell migration assays

Ibidi Culture Inserts (DKSH, Sydney, Australia) were placed in the center of 6 well plates. BE(2)-C cells were added into the wells outside the inserts. Sixteen hours later, the inserts were removed to create “wounds”. Photos of the “wounds” were taken under microscope, and the areas of the remaining “wounds” were measured and analyzed with Image J software (National Institutes of Health, USA).

### Cell invasion assays

BE(2)-C cells were transfected with siRNA or treated with JMJD1A inhibitors for 20 hours, and then detached and added onto BD Falcon Cell Culture Inserts coated with Matrigel inside BD BioCoat Matrigel Invasion Chambers (Becton Dickinson, Bedford, MA, USA). Cells invaded through Matrigel to the bottom side of the inserts were stained with toluidine blue, photographed under microscope and quantified.

### ChIP assays

ChIP assays were performed with an anti-N-Myc, anti-JMJD1A, anti-di-methyl H3K9 or control antibody and PCR with primers targeting up-stream negative control region or core promoter region of the JMJD1A or MALAT1 gene promoter with the protocol we described [38]. Fold enrichment of the JMJD1A or MALAT1 gene core promoter by the anti-N-Myc, anti-JMJD1A or anti-di-methyl H3K9 antibody was calculated by dividing PCR products from the gene core promoter region by PCR products from the up-stream negative control region.

### Histone demethylase inhibition assays

The inhibitory activities of NOG against JMJD1A were assayed according to a previously reported method [39]. The inhibitory activity of NOG against JMJD2A and JMJD2C were assayed according to the method we reported previously [40]. The JARID1A activity after treatment with vehicle control or NOG was measured by the formaldehyde dehydrogenase-coupled assay as described for JMJD2A and JMJD2C, except that reactions were performed with H3K4me3 peptide in a final volume of 30 μL in 384-well plate and a final concentration of JARID1A was 0.64 mg/mL.

### Statistical analysis

All data for statistical analysis were calculated as mean ± standard error, and differences were analyzed for significance using ANOVA among groups or unpaired t-test for two groups. A probability value of 0.05 or less was considered significant.

## SUPPLEMENTARY FIGURE AND TABLES




