# Supplementary material for: The histone demethylase JMJD1A induces cell migration and invasion by up-regulating the expression of the long noncoding RNA MALAT1
Source: Oncotarget. 2014 Feb 18;5(7):1793–804. doi: 10.18632/oncotarget.1785 (PMC4039110; doi:10.18632/oncotarget.1785)
Supplement: Supplementary file 1 [file oncotarget-05-1793-s001.pdf]

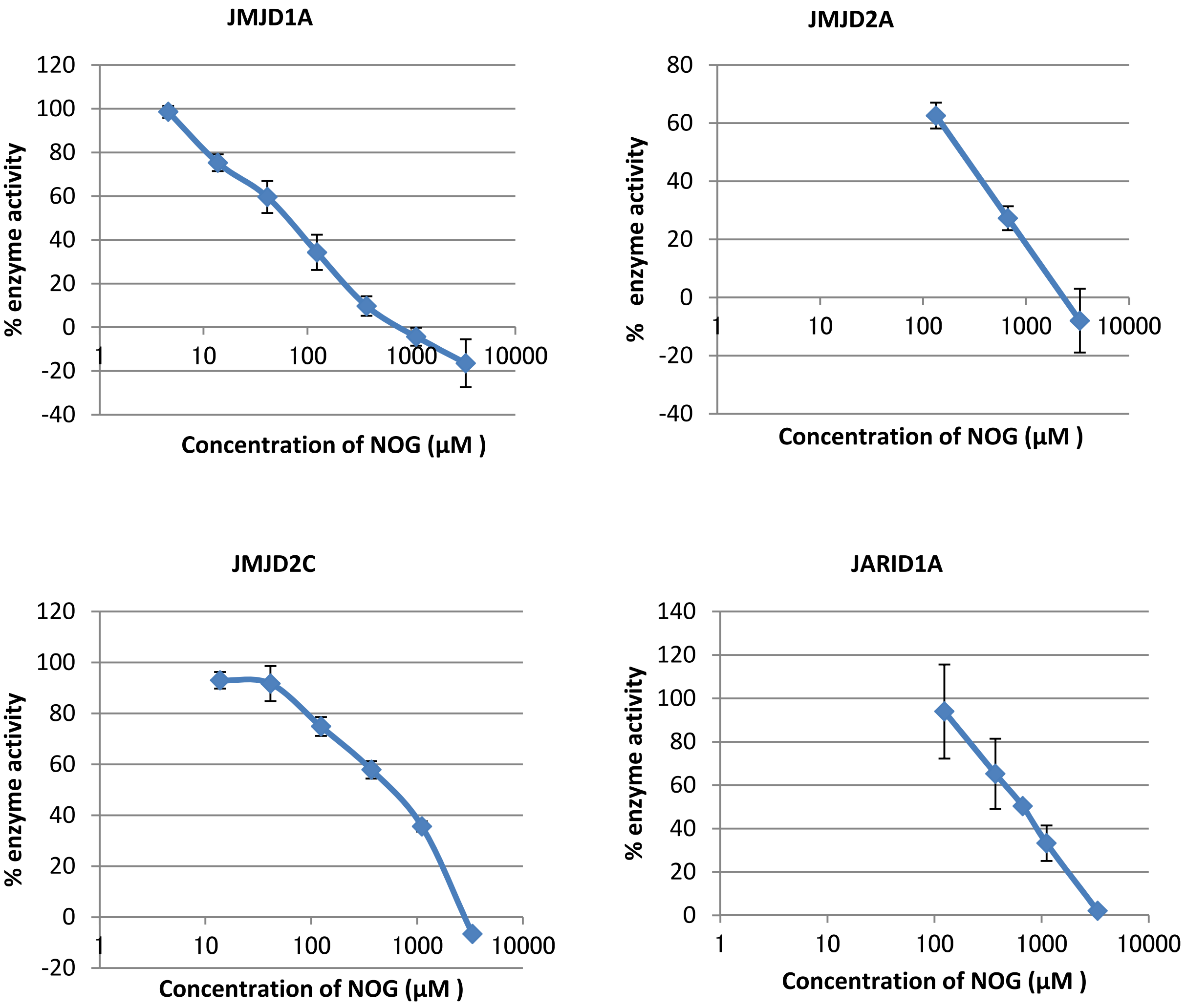

Figure S1: The half maximal inhibitory concentrations (IC_{50}) of *N*-oxalylglycine (NOG) against JMJD1A, JMJD2A, JMJD2C and JARID1A *in vitro*. The assays were described in details in Methods.
